# Implementation of a provider-focused intervention for maximizing human papillomavirus (HPV) vaccine uptake in young cancer survivors receiving follow-up care in pediatric oncology practices: protocol for a cluster-randomized trial of the HPV PROTECT intervention

**DOI:** 10.1186/s12887-022-03562-1

**Published:** 2022-09-12

**Authors:** Wendy Landier, Smita Bhatia, Joshua S. Richman, Paula D. Campos Gonzalez, Brooke Cherven, Veronica Chollette, Jamie Aye, Sharon M. Castellino, Maria M. Gramatges, Susan Lindemulder, Thomas B. Russell, Lucie M. Turcotte, Graham A. Colditz, Melissa B. Gilkey, James L. Klosky

**Affiliations:** 1 Institute for Cancer Outcomes and Survivorship, University of Alabama at Birmingham, 1600 7th Ave. South, Lowder 500, Birmingham, Alabama, 35233 USA; 2 Department of Pediatrics, Division of Pediatric Hematology/Oncology, University of Alabama at Birmingham, 1600 7th Ave. South, Lowder 512, Birmingham, Alabama, 35233 USA; 3 Department of Pediatrics, Emory University and Children’s Healthcare of Atlanta, 2015 Uppergate Drive, ECC#412, Atlanta, GA 30322 USA; 4 Healthcare Systems and Interventions Research Branch, Healthcare Delivery Research Program, Division of Cancer Control and Population Sciences, National Cancer Institute, 9609 Medical Center Dr., Room 3E344, MSC 9762, Rockville, MD 20850 USA; 5 Department of Pediatrics, Division of Pediatric Hematology/Oncology, Baylor College of Medicine, 1102 Bates St., Suite 1200, Houston, TX 77030 USA; 6 Department of Pediatrics, Oregon Health & Science University, 3181 SW Sam Jackson Park Road, Portland, Oregon, 97239 USA; 7 Department of Pediatrics, Wake Forest University, Medical Center Boulevard, Winston-Salem, North Carolina 27157 USA; 8 Department of Pediatrics, University of Minnesota, D-557 Mayo Building, 420 Delaware Street SE, Minneapolis, MN 55455 USA; 9 Department of Surgery, Washington University at St. Louis School of Medicine, 660 S. Euclid Ave, St. Louis, MO 63110 USA; 10 Department of Health Behavior, Gillings School of Global Public Health, University of North Carolina, 317 Rosenau Hall, CB #7440, 135 Dauer Drive, Chapel Hill, North Carolina 27599 USA

**Keywords:** Human papillomavirus, Vaccination rates, Childhood cancer survivors, Cluster-randomized trial

## Abstract

**Background:**

Childhood cancer survivors are at high risk for developing new cancers (such as cervical and anal cancer) caused by persistent infection with the human papillomavirus (HPV). HPV vaccination is effective in preventing the infections that lead to these cancers, but HPV vaccine uptake is low among young cancer survivors. Lack of a healthcare provider recommendation is the most common reason that cancer survivors fail to initiate the HPV vaccine. Strategies that are most successful in increasing HPV vaccine uptake in the general population focus on enhancing healthcare provider skills to effectively recommend the vaccine, and reducing barriers faced by the young people and their parents in receiving the vaccine. This study will evaluate the effectiveness and implementation of an evidence-based healthcare provider-focused intervention (HPV PROTECT) adapted for use in pediatric oncology clinics, to increase HPV vaccine uptake among cancer survivors 9 to 17 years of age.

**Methods:**

This study uses a hybrid type 1 effectiveness-implementation approach. We will test the effectiveness of the HPV PROTECT intervention using a stepped-wedge cluster-randomized trial across a multi-state sample of pediatric oncology clinics. We will evaluate implementation (provider perspectives regarding intervention feasibility, acceptability and appropriateness in the pediatric oncology setting, provider fidelity to intervention components and change in provider HPV vaccine-related knowledge and practices [e.g., providing vaccine recommendations, identifying and reducing barriers to vaccination]) using a mixed methods approach.

**Discussion:**

This multisite trial will address important gaps in knowledge relevant to the prevention of HPV-related malignancies in young cancer survivors by testing the effectiveness of an evidence-based provider-directed intervention, adapted for the pediatric oncology setting, to increase HPV vaccine initiation in young cancer survivors receiving care in pediatric oncology clinics, and by procuring information regarding intervention delivery to inform future implementation efforts. If proven effective, HPV PROTECT will be readily disseminable for testing in the larger pediatric oncology community to increase HPV vaccine uptake in cancer survivors, facilitating protection against HPV-related morbidities for this vulnerable population.

**Trial registration:**

ClinicalTrials.gov Identifier: NCT04469569, prospectively registered on July 14, 2020.

## World Health Organization trial registration data set

**Table:** 

Data category	Information
Primary registry and trial identifying number	ClinicalTrials.gov NCT04469569
Date of registration in primary registry	14 July 2020
Secondary identifying numbers	UAB IRB-300005305
Source(s) of monetary or material support	U.S. National Cancer Institute U01CA246567 (PIs WL and JLK)
Primary sponsor	U.S. National Cancer Institute
Secondary sponsor(s)	University of Alabama at Birmingham
Contact for public queries	Wendy Landier, PhD wclandier@uabmc.edu
Contact for scientific queries	Wendy Landier, PhD wclandier@uabmc.edu
Public title	Provider-Focused Intervention for Maximizing HPV Vaccine Uptake in Young Cancer Survivors
Scientific title	Implementation of a Provider-Focused Intervention for Maximizing HPV Vaccine Uptake in Young Cancer Survivors receiving Follow-Up Care in Pediatric Oncology Practices: A Cluster-Randomized Trial
Countries of recruitment	United States
Health condition(s) or problem(s) studied	Prevention of HPV-related subsequent neoplasms in childhood cancer survivors
Intervention(s)	HPV PROTECT intervention vs. usual care
Key inclusion and exclusion criteria	*Childhood cancer survivors:* 9-17y of age; ≥ 1y following completion of cancer therapy; reside in state where targeted clinic is located; receiving follow-up care (in person or via telehealth) at participating site. *Healthcare providers:* ≥ 18y of age; provide care for childhood cancer survivors meeting inclusion criteria; licensed to order vaccines; willing to complete study surveys and/or interviews.
Study type	Interventional (clinical trial)
Date of first enrolment	1 Feb 2021
Target sample size	5196
Recruitment status	Recruiting
Primary outcome(s)	HPV vaccine initiation rates for childhood cancer survivors, age 9-17y and ≥ 1y post-completion of cancer therapy
Key secondary outcomes	Healthcare provider perspectives regarding intervention feasibility, acceptability, appropriateness, and fidelity; change in healthcare provider HPV vaccine-related knowledge and practices following implementation of the HPV PROTECT intervention; HPV vaccine series completion rates among childhood cancer survivors; sustainability of HPV vaccine initiation rates.

## Background

Childhood cancer survivors are at high risk for developing new cancers (such as cervical, vulvar, vaginal, penile, anal, and oropharyngeal cancer) caused by persistent infection with the human papillomavirus (HPV) [1]. Compared with the age- and sex-matched general population, female and male cancer survivors have a 1.4- to 2.5-fold excess risk, respectively, of developing HPV-related malignancies [2]. Fortunately, HPV-related malignancies are largely preventable due to availability of the nonavalent HPV vaccine [3], which offers protection against ~ 90% of oncogenic HPV subtypes [4, 5]. We have previously shown that uptake of the HPV vaccine is significantly lower in young cancer survivors compared with general population peers (22.0% *vs*. 42.5% in those age 13–17 years), and that lack of healthcare provider recommendation is the strongest predictor of HPV vaccine non-initiation in cancer survivors [6]. We have also shown that the HPV vaccine is safe in young cancer survivors, and that immunogenicity of the 3-dose HPV vaccine series is similar to that seen in the general population [7].

Young people with chronic health conditions, including cancer survivors, often identify their subspecialty provider (i.e., oncologist) as their main healthcare provider [8], have greater confidence in healthcare recommendations made by their oncology providers as compared with primary care providers (PCPs) [9], and may forgo routine primary care services. This may result in receipt of fragmented medical care and unmet healthcare needs, including under-vaccination [8, 10]. Targeting an intervention to pediatric oncology practices provides an opportunity to address low HPV vaccine uptake in childhood cancer survivors, particularly since return of young people to primary care settings for receipt of preventive care has been identified as a barrier to HPV vaccine initiation in the general [11] and subspecialty [12, 13] populations, and survivors regularly return to pediatric oncology clinics for disease-surveillance and survivorship-directed care [9, 14].

Strategies that are most successful in increasing HPV vaccine uptake in the general population focus on enhancing the skills that healthcare providers need to effectively recommend the vaccine, and reducing barriers faced by young people and their parents to receiving the vaccine [15, 16]. Successful interventions to improve HPV vaccine uptake in the pediatric/adolescent general population have included healthcare provider communication training [17–19], provider assessment and feedback [20–24], and tools/resources to engage providers in implementation of practice-level changes that positively impact adolescent HPV vaccine uptake [22, 25, 26]. These existing evidence-based interventions offer a strong foundation for working with providers to improve HPV vaccine uptake, with the caveat that they were developed for use in primary care [17, 20, 23, 24, 27–31]. Adaptation is needed to support their implementation in the oncology settings that serve young cancer survivors.

In this study, we will test the effectiveness of a package of evidence-based intervention materials [17–26], adapted for use by pediatric oncology providers (HPV PROTECT: Healthcare ProVider intervention PROmoting HPV vaccination among TEens after Cancer Treatment), in improving HPV vaccination rates among young cancer survivors, age 9 to 17, and evaluate its implementation in the context of cancer survivorship care.

**Fig. 1:**
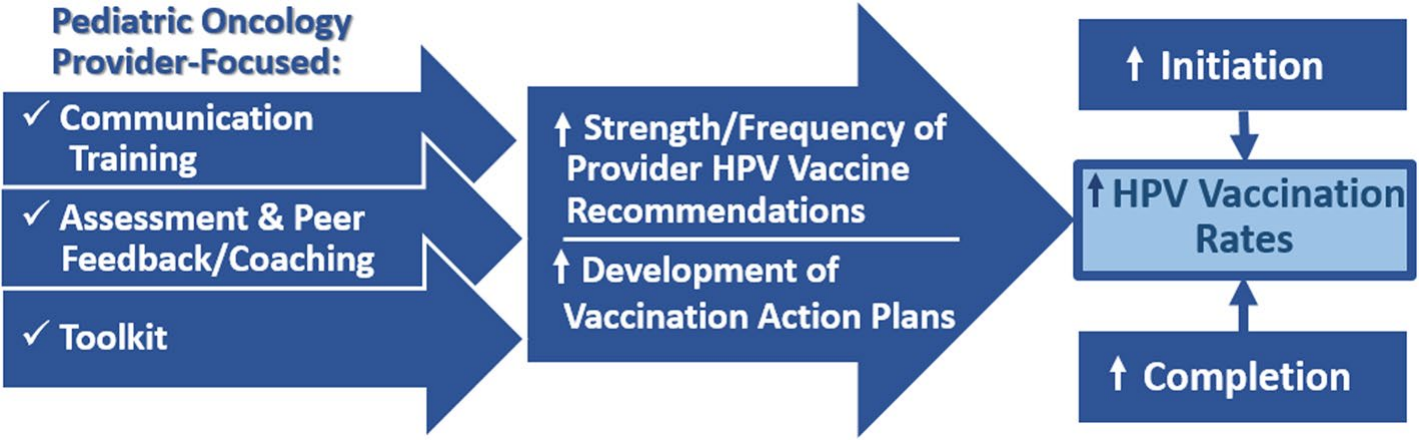
HPV PROTECT intervention

The HPV PROTECT intervention is comprised of three components i) *Provider Communication Training*, designed to increase the quality of provider recommendations as well as the proportion of patients who receive the high quality recommendations for the HPV vaccine [32]; ii) *Assessment and Peer Feedback/Coaching*, which delivers current vaccination rates and clinic goals, as well as highlights techniques to meet those goals [22, 31], and iii) *Provider Toolkit*, which provides practice-focused resources to promote HPV vaccination [22, 26]. Thus, HPV PROTECT is designed to increase provider knowledge regarding use of the HPV vaccine in the cancer survivor population, enhance provider skills in delivering brief, compelling HPV vaccine recommendations to parents of young cancer survivors, present ongoing feedback to providers regarding survivor HPV vaccination rates, and decrease barriers to receipt of vaccine by survivors through the provision of Vaccine Action Plans (Fig. 1).

We will monitor vaccination rates at participating sites throughout the study using state vaccine registries. State vaccine registries (also known as Immunization Information Systems) are confidential, population-based, electronic data repositories across the United States (U.S.) for storage of vaccination information from multiple sources (e.g., health departments, hospitals, clinics, pharmacies and private practices) [33]. The registries are coordinated by the Centers for Disease Control, which maintains Functional Standards, Core Data Elements, and Technical Guidance for these systems within the U.S. [33]. Vaccine registry data have been used in numerous studies to assess outcomes of interventions designed to increase immunization uptake [17, 20, 34–37] and have been shown to be reliable when compared with medical records and parental report [38]. Thus, state vaccine registries are a robust source for assessing vaccine uptake, regardless of where survivors receive their vaccinations within the state [33, 39, 40].

This study will use a hybrid type 1 effectiveness-implementation approach [41, 42]. Effectiveness of the HPV PROTECT intervention will be evaluated in a cluster-randomized, stepped-wedge trial [43] over 4 years, with a multi-state sample of pediatric oncology clinics that provide care to cancer survivors within the targeted age (9–17 years) and time frame (≥ 1 year off-therapy) for vaccination [44, 45]. The study will simultaneously evaluate implementation outcomes (provider perspectives regarding feasibility, acceptability and appropriateness of HPV PROTECT in pediatric oncology clinics, intervention fidelity, and change in healthcare provider HPV vaccine-related knowledge and practices) to inform future implementation strategies.

## Objectives

### Primary objective

Among childhood cancer survivors 9–17 years of age and ≥ 1 year post-completion of cancer therapy (targeted age/time frame for vaccination) who are returning for follow-up care (in-person or via telehealth) across six geographically-diverse pediatric oncology clinics, conduct a cluster-randomized, stepped-wedge trial to evaluate the effectiveness of HPV PROTECT in improving HPV vaccine initiation rates one year post-intervention. We hypothesize that implementation of HPV PROTECT will significantly increase HPV vaccine initiation rates in survivors of childhood cancer.

### Secondary objectives

Ascertain healthcare provider perspectives regarding feasibility, acceptability and appropriateness of HPV PROTECT in the pediatric oncology setting; assess intervention fidelity; and estimate change in healthcare provider HPV vaccine-related knowledge and practices.

### Exploratory objectives

Estimate the effect of HPV PROTECT on: i) HPV vaccine series completion; and ii) ongoing improvement in HPV vaccine initiation rates in the years subsequent to HPV PROTECT implementation (sustainability).

## Implementation framework

This study will employ the RE-AIM (Reach, Effectiveness, Adoption, Implementation, Maintenance) implementation framework [46, 47] to evaluate implementation outcomes (i.e., process evaluation) [48]. Data for this evaluation will be collected simultaneously with the effectiveness trial to inform future implementation efforts. A mixed-methods approach (surveys and qualitative interviews) will be used to conduct the outcomes evaluation, with questions and tools/methods guided by the RE-AIM framework.

## Methods

### Ethics approval

The study described here was approved by the University of Alabama at Birmingham (UAB) Institutional Review Board (IRB-300005305), with UAB serving as the single IRB (sIRB) of record. In accordance with the SMART (Streamlined, Multisite, Accelerated Resources for Trials) IRB reliance model, participating sites ceded review to the UAB IRB as the Reviewing IRB under the Master Common Reciprocal IRB Authorization Agreement.

### Study design, setting, and population

**Fig. 2:**
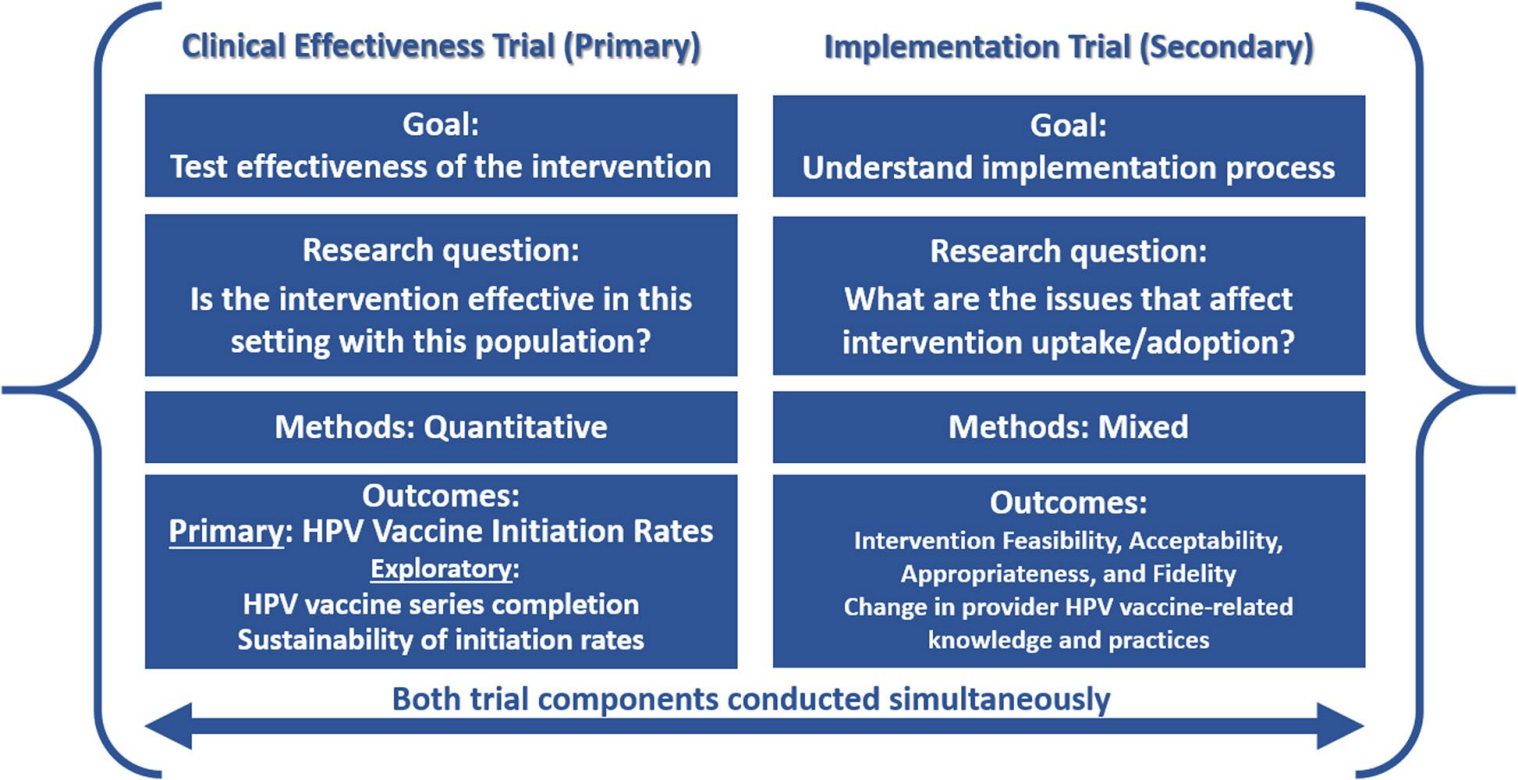
Hybrid type 1 effectiveness-implementation trial design

A hybrid type 1 effectiveness-implementation design will be used to test the effectiveness of the HPV PROTECT healthcare provider-directed intervention in improving HPV vaccine uptake in childhood cancer survivors, and to evaluate implementation outcomes (Fig. 2)*.* This study will be conducted at six participating sites (The University of Alabama at Birmingham/Children’s of Alabama, Emory University/Children’s Healthcare of Atlanta, Baylor College of Medicine/Texas Children’s Hospital, Oregon Health & Science University/Doernbecher Children’s Hospital, Atrium Health Wake Forest Baptist, and the University of Minnesota/M Health Fairview Masonic Children’s Hospital). Each of these sites have active pediatric oncology outpatient clinics that provide ongoing care for young cancer survivors 9–17 years of age and at least 1 year off-therapy, and each clinic has existing access to their state vaccine registry via their electronic medical record (EMR) system. Sites were chosen to be representative of pediatric oncology practices of varying characteristics including varied geographic location, size, and patient populations, and were especially enriched for sites serving populations in areas of lowest HPV vaccine uptake (i.e., U.S. southern states) [49, 50]. None of these sites have a standardized (quality improvement) program in place to address HPV vaccine initiation in cancer survivors. Each site has an identified Intervention Champion, who will be responsible for all aspects of the study at their site, including overseeing data collection, engaging and training providers in the targeted pediatric oncology clinics, identifying context-specific resources, assuring availability and context-appropriateness of intervention resources, providing ongoing group peer feedback/coaching sessions to providers, overseeing interface with clinic support staff in carrying out all aspects of the intervention, and assuring intervention fidelity. Each participating site has agreed to operationalize HPV PROTECT as a quality improvement (QI) initiative; therefore, all providers caring for cancer survivors will be expected to complete the training sessions, and will be given access to all intervention components at the time of intervention implementation. Healthcare providers at the participating sites will be invited, but not required, to participate in the research aspects of the project.

### HPV PROTECT intervention components

#### Provider communication training

This training session, presented to clinicians at each site by the Intervention Champion, is based on the Announcement Approach Training framework [17, 20, 32]. Content of the program is tailored to the pediatric oncology provider caring for young cancer survivors, and includes survivor-specific information, such as timing of vaccination post-therapy, and number of doses needed to complete the vaccine series [7]. Thus, this training is designed to increase provider knowledge of survivor-specific HPV vaccine issues and to enhance provider skills in recommending HPV vaccine to young cancer survivors and their parents.

#### Assessment and peer feedback/coaching

This training session, presented to clinicians at each site by the Intervention Champion, includes review of specific evidence related to risk of HPV-related cancers in childhood cancer survivors, the low vaccine uptake among survivors, and the specific HPV vaccination rates among 9–17-year-old cancer survivors at the site (using Vaccine Report Card data). The Intervention Champion will also describe the HPV PROTECT intervention components and resources, identify the site’s goal for improving HPV vaccine initiation rates for survivors, and highlight the techniques and tools that will be employed toward meeting the goal [20–22, 31].

#### Provider toolkit

This toolkit will be introduced by the Intervention Champion and made available to clinicians at each site during the Intervention Year. The Provider Toolkit includes practice-focused resources designed to facilitate HPV vaccine uptake in the pediatric oncology setting [22, 26], as follows: *i) Vaccine Action Plan* for providers to complete with parents, tailored to local context. For example, the Vaccine Action Plan will include details regarding where the survivor can initiate and/or complete the vaccine series, if not feasible in the pediatric oncology setting (e.g., PCP office, health department, pharmacy) based on local context and site-specific practices; *ii) Provider Recommendation Note Cards*, which summarize key messages for recommending the HPV vaccine to survivors, as presented in the communication training session; *iii) Survivor Vaccine Fact Sheets*, which highlight pediatric oncology-specific information regarding the HPV vaccine for survivors; *iv) Standardized templates for oncologist-PCP communication*, regarding the vaccination plan; *v) Standardized templates for oncologist-parent communication* regarding the agreed-upon plan for vaccination, for inclusion in clinic discharge paperwork; *vi) EMR Documentation Template*, for documenting discussion of the HPV vaccine recommendation and plan for vaccine receipt in the EMR; and vii) *HPV PROTECT Intervention Website*, which provides access to intervention resources and video-recordings of training sessions for providers at participating sites. All HPV PROTECT components are designed to be accessible via this web-based platform. All participating study sites will have access to the HPV PROTECT website, with controlled release concurrent with the intervention year.

### Adaptation of the intervention to local context

Intervention Champions will be given the opportunity to adapt components of the Provider Toolkit (e.g., Vaccine Action Plan; Recommendation Videos), in order to add salient information that may be necessary for survivors to procure the vaccine locally, as well as to adapt training videos to feature local influential providers.

### Intervention fidelity

To assure intervention fidelity, all components of the intervention will be standardized and manualized prior to delivery. The site Intervention Champions will undergo intensive intervention-specific training (including a 2-day intensive training group program followed by individual meetings) led by the study PIs and co-investigators with intervention-related expertise, beginning two to three months prior to intervention implementation at their sites. This train-the-trainer approach includes review of all aspects of the intervention, use of the intervention manual, training in skill sets relevant to leading the intervention and supporting providers at their sites, and assistance in adapting intervention materials to local context. The site Intervention Champions will be responsible for monitoring all aspects of fidelity at their sites and for completion of a fidelity checklist. Toolkit resources will be tailored to site-specific local context as specified in the intervention manual; however, all training sessions for healthcare providers, including those adapted for local context, will remain scripted, regardless of the individual delivering the messaging. Thus, training sessions delivered by the site Intervention Champions, will incorporate standardized scripts and presentation materials, and will be observed by the study Principal Investigators (PIs) for fidelity. Intervention Champions will monitor provider participation in intervention training sessions and ongoing use of intervention components (including Toolkit resources) at their sites, and will track participation in peer feedback meetings. Site Intervention Champions and study PIs will meet at least quarterly to address any issues or challenges that may arise.

### Participants

#### Healthcare providers

Healthcare providers are the main target for this implementation trial, and the provider-directed intervention occurs at the clinic level; thus, the research component of the trial focuses on the providers. Eligible providers will be invited to participate in the research study in order to assess their knowledge and current practices related to the HPV vaccine in young cancer survivors, and to understand provider perspectives regarding the feasibility, acceptability and appropriateness of the HPV PROTECT intervention components, and provider adherence (fidelity) to each of the intervention components.

#### Childhood cancer survivors

The population of interest for measurement of HPV vaccine initiation and completion rates is defined as: Childhood cancer survivors, who are 9 to 17 years of age and at least 1-year following completion of cancer therapy, and are returning to one of the targeted pediatric oncology clinics for follow-up care.

### Recruitment

#### Healthcare providers

Potential participants for this study will be identified using rosters of eligible healthcare providers, and their associated email addresses, at participating sites (compiled by the site Intervention Champions). Each site Intervention Champion will send all eligible healthcare providers at their site an email introducing the study coordinating center and describing study procedures, including risks and benefits of participation. The study coordinating center will then send each eligible healthcare provider an invitation to participate in the study.

#### Childhood cancer survivors

Childhood cancer survivor vaccine initiation and completion rates will be measured as part of each site’s QI initiative to increase HPV vaccination rates. No patient/parent recruitment will be conducted by the sites, since patients/parents will be only indirectly involved in the research through the calculation of clinic-level vaccination rates, and a full Health Information Portability and Accountability (HIPAA) waiver is in place. All patient-level data collected for this study will be completely de-identified (removal of the 18 protected health information [PHI] identifiers), prior to submission to the study coordinating center.

### Inclusion and exclusion criteria

#### Healthcare providers

*Inclusion:* Healthcare providers (physicians, advanced practice providers) in the targeted pediatric oncology clinics at the participating sites: i) caring for childhood cancer survivors; ii) licensed to order vaccines; iii) willing to complete surveys and/or interviews. *Exclusion:* Meets eligibility criteria but unwilling to provide informed consent for study participation.

#### Childhood cancer survivors

*Inclusion:* Childhood cancer survivors who are 9–17 years of age, ≥ 1 year following completion of cancer therapy, receive follow-up care (in person or via telehealth) in a targeted pediatric oncology clinic at a participating site, and reside in the state where the clinic is located. *Exclusion:* State vaccine registry data not available for the survivor.

### Informed consent

#### Healthcare providers

Healthcare providers who provide care to patients in the targeted population will receive a study email from the coordinating center with an information sheet, along with a link to the online survey. The IRB has authorized waiver of consent documentation; thus, completion of the survey will imply consent. An item on the survey will ask the healthcare provider if they wish to participate in study interviews. Those indicating interest in interview participation will be contacted by the study Clinical Research Assistant (CRA) at the study coordinating center via email or telephone. Verbal consent will be obtained prior to interview initiation.

#### Childhood cancer survivors

There will be no interaction between the study team and cancer survivors. Study involvement includes abstraction of existing clinical information only.

### Approach

#### Effectiveness trial

The HPV PROTECT intervention will be implemented as standard of care (quality improvement), and will be evaluated across the six participating sites using a cluster-randomized stepped-wedge design. During the first year of the trial, all six participating sites will be assigned to the baseline (control) condition in which they will proceed with usual care and measure HPV vaccination rates of the targeted population (cancer survivors 9–17 years of age and ≥ 1 year following completion of therapy) using state vaccine registry data accessible via EMRs. In Year 2, three of the sites will implement *HPV PROTECT* while the remaining three sites will continue with usual care. In Year 3, the remaining three sites will implement *HPV PROTECT,* and sustainability of the intervention will be evaluated for the three sites that implemented *HPV PROTECT* in Year 2. In Year 4, sustainability (i.e., ongoing improvement in vaccine initiation rates) of the *HPV PROTECT* intervention will be evaluated across all six sites. Measurement of HPV vaccination rates using state vaccine registry data accessible via EMRs will continue across the entire 4-year period of the trial.

#### Implementation trial

To inform future dissemination of the intervention, we will use a mixed methods approach to estimate change in healthcare provider HPV vaccine-related knowledge and practice; evaluate healthcare provider perspectives regarding intervention feasibility, acceptability, and appropriateness in the pediatric oncology setting; and evaluate provider adherence (fidelity) to intervention components.

All of the targeted pediatric oncology healthcare providers at each clinic will be invited to complete electronically-administered surveys, yearly for four years, via *Research Electronic Data Capture (REDCap®)* [51], a secure password-protected, web-based research database, regarding their HPV vaccine-related knowledge, current HPV vaccine-related practices (e.g., provision of vaccine recommendations to parents/patients), their evaluation of the HPV PROTECT intervention components, and their sociodemographic information (e.g., age, sex, education, role, years of experience). Healthcare providers who complete surveys will be asked to indicate if they would like to participate in qualitative interviews in the survey year during which the participant’s site is implementing the HPV PROTECT intervention. Healthcare providers who indicate interest in interview participation will be invited to participate in telephone or secure web-based interviews regarding feasibility and acceptability of the intervention components and barriers/facilitators to implementation of the intervention. Interviews will be recorded and transcribed by the coordinating center CRA. Personal identifiers will be removed from the data by the CRA following data collection. Results will be reported in aggregate without personal identifiers.

### Randomization

**Fig. 3:**
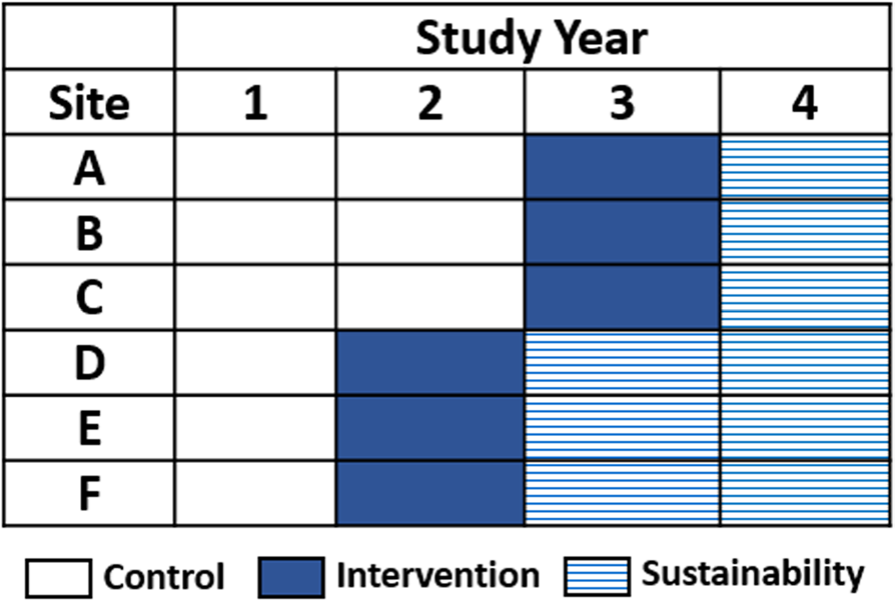
Stepped-wedge cluster-randomized trial design

**Fig. 4:**
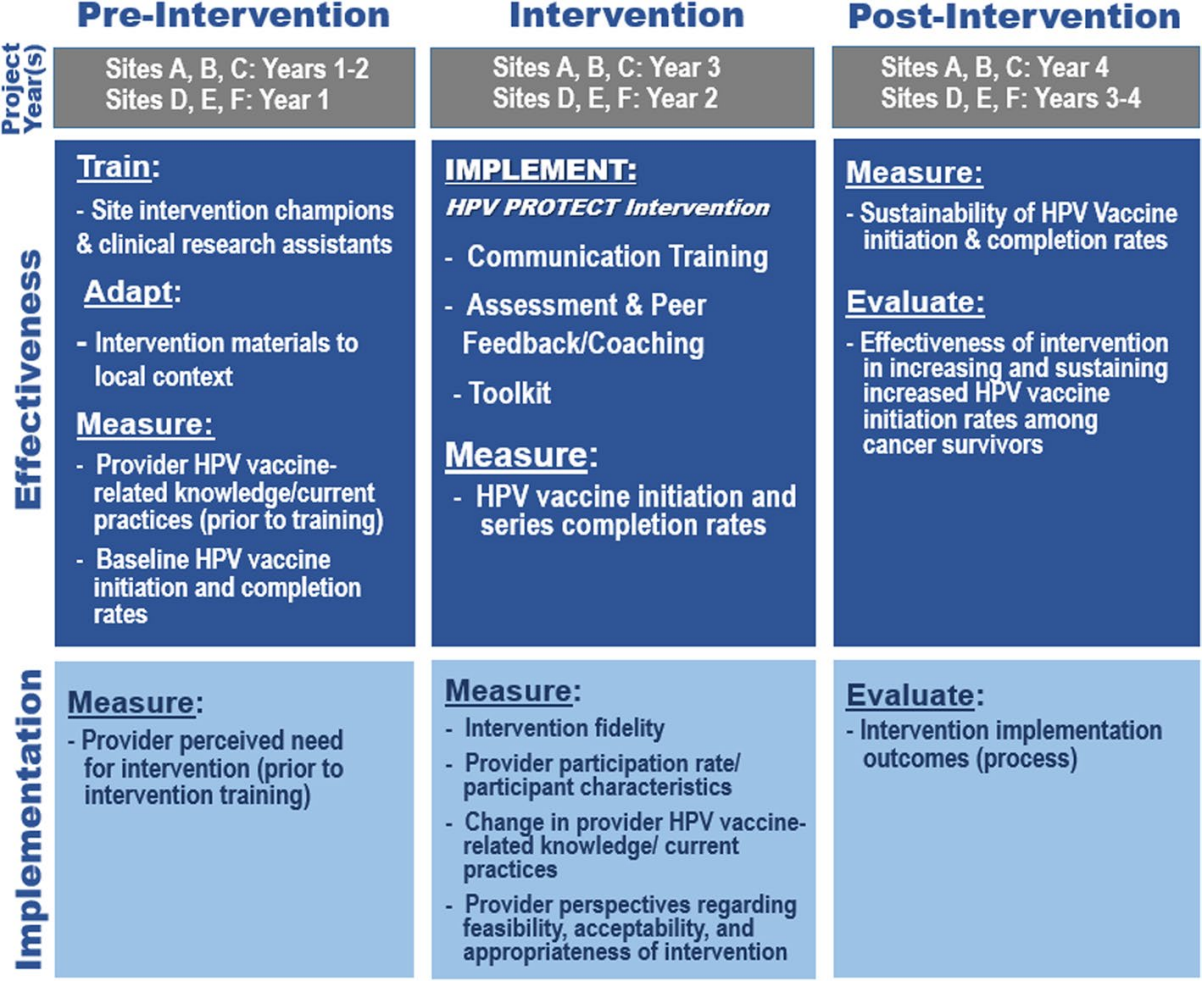
Study schema

In this stepped-wedge cluster-randomized design, randomization will occur at the site level, with three sites randomized to begin the intervention in Year 2, and three sites randomized to begin the intervention in Year 3 (Fig. 3). There is no difference in the intervention between the two groups other than the intervention start date. The study’s stepped-wedge design incorporates a control period for all participating sites during Year 1, allowing for collection of baseline vaccination rates, thus avoiding historical comparisons. Before the beginning of Year 2, sites will be randomized according to the stepped-wedge design. The sites assigned to the control condition in Year 2 (sites A, B, C) will be assigned to the intervention in Year 3, and the Year 2 intervention sites (sites D, E, F) will be moved to the post-intervention (sustainability) condition for Year 3. All sites will be assigned to the post-intervention (sustainability) condition for Year 4. The stepped wedge design will allow comparison of the primary outcome (HPV vaccine initiation rate; i.e., proportion of survivors in targeted population who have received ≥ 1 HPV vaccine dose, as of the last day of each study year) across groups (control vs. intervention), while allowing each site to serve as its own pre-intervention control, and using the control observations in Years 1 (all sites) and 2 (sites A, B, C) to estimate secular trends. Sustainability will be assessed in the post-implementation period, i.e., in Years 3 (sites D, E, F) and 4 (all sites) (Fig. 4).

### Measures

#### Healthcare provider data

Participating healthcare providers will complete the following:

*Healthcare Provider Electronic Survey*. The healthcare provider survey will be completed electronically via *REDCap®* yearly for 4 years in Years 1–4; estimated completion time is 15 min per year. The survey will ascertain the following data: Sociodemographics (age, sex, race/ethnicity, education); Clinical characteristics (provider license type [MD, NP, PA]; role [attending, staff, trainee], years of experience in current role, average number of survivors seen per week); provider HPV vaccine-related knowledge/practices (knowledge regarding HPV vaccine, familiarity with strategies to increase vaccine uptake, recall [or prior knowledge] of key messages in the training program, current practices related to the HPV vaccine (e.g., method used to assess survivor vaccination status, frequency and quality of recommendation for HPV vaccine, strategies used to promote HPV vaccination); and intervention evaluation (objective rating of feasibility, acceptability, appropriateness of the intervention in the pediatric oncology setting and provider fidelity to intervention components [e.g., vaccine recommendations, educational materials]).

*Healthcare Provider Qualitative Interview.* Semi-structured qualitative interviews will be conducted with those healthcare providers who opt-in to this portion of the study. The interviews will occur once, during the year that the healthcare provider’s site initiates the intervention; estimated interview duration is 30 min. Providers will be asked about their perceptions of the feasibility (perceived potential for success), acceptability (perceived need), and appropriateness (perceived fit) of the intervention to the pediatric oncology setting, and their adherence (fidelity) to intervention components (e.g., delivery of recommendations, developing and communicating vaccine action plans to parents and PCPs). Interviews will be conducted during the second half of Year 2 (sites D, E, F) and Year 3 (Sites A, B, C) via telephone or secure web-based platform by an interviewer from the coordinating center trained in qualitative interview techniques. An Interview Guide will be used and interviews will be recorded and transcribed verbatim.

#### Patient-level data

The intervention occurs at the clinic level, and medical record and vaccine registry data for the cancer survivor population will be accessed by the site CRA under a HIPAA waiver as part of each site’s QI initiative. The site CRA will abstract the following data elements from the medical record for each survivor added to the study roster: Diagnosis category (leukemia, lymphoma, solid tumor, central nervous system [CNS] tumor); age at diagnosis, time from diagnosis, and time off-therapy at cohort entry; history of hematopoietic cell transplant (yes/no; if yes: type, number, age); history of Chimeric Antigen Receptor (CAR)-T cell therapy (yes/no: If yes: number, age); sex; and race/ethnicity. The CRA will abstract the following additional visit-specific data elements from the survivor’s medical record for the initial clinic visit and for each subsequent clinic visit occurring during the study period: Age at visit, study year and week of visit, provider type (physician, advanced practice provider), study identification number of healthcare provider(s) providing care at clinic visit, visit type (in-person, telehealth), and insurance type.

Upon adding each survivor to the study roster, the CRA will access the state vaccine registry and abstract all HPV vaccine dose(s) and associated date(s) of administration occurring prior to the date of the survivor’s cohort entry visit. At least yearly throughout the data collection period (Years 1–4), the CRA will access the state vaccine registry and abstract any additional HPV vaccine dose(s) and associated date(s) of administration occurring since the most recent data abstraction. Additionally, dose(s) and date(s) of additional vaccines recommended for adolescents (i.e., meningococcal, Tdap, influenza and COVID-19), will be abstracted by the CRAs at the same timepoints, for use as co-variates in the analysis, as it is important to understand whether uptake of the HPV vaccine differs from uptake of the other recommended adolescent vaccines [52]. All data will be completely de-identified prior to submission to the study coordinating center; survivors with visits in more than one year will be linked so that multiple observations per survivor can be addressed in the analysis. Coded roster data (with no elements of personal health information [PHI]) will be submitted weekly by participating sites to the study coordinating center via the study’s REDCap® study database. Data quality will be monitored in real-time by the site CRAs for each individual site and by the CRA at the study coordinating center for the overall study, using the study Standardized Operating Procedures, which include detailed instructions for data collection, verification, and cleaning. Additionally, range checks for key variables, and warnings to prevent missing data, are built into the REDCap® study database.

### Outcomes

#### Primary outcome

Change in survivor HPV vaccine initiation rates one year following intervention implementation, measured using state vaccine registry data accessed via the EMR at each participating site as part of the QI project associated with this research. The primary outcome will be measured as of the final day of the intervention year (Year 2 for sites DEF, Year 3 for sites ABC).

#### Secondary outcomes

Healthcare provider knowledge and current practices related to the HPV vaccine, measured by survey (yearly in Years 1–4). Provider perspectives regarding feasibility/acceptability and appropriateness of the intervention in the pediatric oncology setting, and provider adherence (fidelity) to intervention components, measured by survey (yearly in Years 1–4), and interview (once during intervention year [Year 2 or Year 3]).

#### Exploratory outcomes

Change in survivor HPV vaccine series completion rates, and ongoing HPV vaccine initiation rates in years subsequent to implementation of the intervention (sustainability), measured using state vaccine registry data accessed via the EMR at each participating site as part of the QI project associated with this research. The exploratory outcomes will be measured as of the final day of each relevant study year.

### Analytic plan

#### Effectiveness trial

Unless otherwise noted, all statistical tests will be two-sided. The main hypothesis of the intervention’s effect will be tested using a mixed effects logistic regression model including a random intercept for each site. The base model for HPV vaccine initiation for participant *i* at site* j* during year* y* without covariates will be:

$$\mathrm{log}\left({Odds}_{initiation,i,j,y}\right)={\beta }_{0}+{\beta }_{1}\bullet {year}_{j,y}+{\beta }_{2}\bullet {x}_{intervention,j,y}+{b}_{j}$$

where ${year}_{i,j}$ indicates the study-year of the visit, and ${x}_{intervention,j,y}$ equals 0 if site *j* has not been exposed to the intervention by ${year}_{j,y}$, and equals 1 if it has, and ${b}_{j}$ is the random intercept for site *j*. The primary hypothesis will be tested by the significance of the coefficient ${\beta }_{2}$ in the model, which will capture the initial year of active intervention. Exploratory aims include testing whether the intervention results in increased rates of series completion and whether there is a sustained intervention effect in increasing HPV vaccine initiation. The analysis for series completion will parallel the analysis for initiation, with the exception that the analytic cohort for each year will be limited to those who had not completed the series prior to their index clinic visit for that year. Sustained effects will be tested using a similar model augmented by specific coefficients for duration of time in the intervention arm:

$$\mathrm{log}\left({Odds}_{initiation,i,j,y}\right)={\beta }_{0}+{\beta }_{1}\bullet {year}_{j,y}+{\beta }_{2}\bullet {x}_{intevention,j,y}+{\beta }_{3}\bullet {x}_{2int,j,y}+{\beta }_{4}\bullet {x}_{3int,j,y}+{b}_{j}$$

In this model, ${\beta }_{1}$ will continue to account for a constant secular trend, while ${\beta }_{3}$ will allow for an additional effect in the 2^nd^ year of intervention and ${\beta }_{4}$ will similarly allow for an additional incremental effect in the third year. Thus, the log odds of initiation for a participant at site *j* in year 4 for a site in the third year of intervention will be estimated as ${\beta }_{0}+{\beta }_{1}\bullet 4+{\beta }_{2}+{\beta }_{3}+{\beta }_{4}$ + ${b}_{j}$. While hypothesis testing will be included in the exploratory aims, the focus will be on reporting estimated rates of outcomes and intervention effects with confidence intervals. For each outcome, secondary analyses will include patient age at visit, sex, insurance type, and race/ethnicity as covariates to improve the precision of estimates and provide an estimated standardized prevalence. We will also evaluate the impact of clinic level factors on vaccination initiation rates (e.g., clinic volume, provider characteristics, practice size, geographic location, and baseline initiation rates) as fixed effects. We will also report the HPV vaccine initiation rate among survivors who were vaccine-naïve at cohort entry (i.e., incidence of vaccine initiation). Last, we will perform subgroup analyses stratified by relevant biological variables (i.e., age at visit, sex, and race/ethnicity groups). For survivors who complete > 1 clinic visit/study year, the number and timing of subsequent clinic visits during the study period will be included as covariates in secondary analyses. Patients who develop a relapse or subsequent malignancy requiring active cancer treatment at any time during the study period will be removed from the data set as of the date of diagnosis of the relapse or subsequent malignancy. Parallel analyses will further consider providers as random effects within sites, adding a random intercept for each provider. Given the potential complexity of correlation structures and nesting, the main hypothesis tests will also be reported using permutation testing. As an example, for permutation testing the main effect based on coefficient ${\beta }_{2}$, for multiple (> 1000) iterations, participants will be randomly assigned to sites and the models fit to develop an empirical distribution for estimates of ${\beta }_{2}$ under the null hypothesis of not different. The p-value of ${\beta }_{2}$ (with genuine assignments) will be estimated from distribution of coefficients estimated from the permutations.

*Sample Size and Power.* Based on a combination of our prior work [6], secular trends since publishing that work, and general population data regarding state-specific vaccination rates [53], pre-intervention prevalence rates of vaccine-initiated patients at the 6 sites are estimated to be 0.404, 0.496, 0.462, 0.473, 0.415, and 0.488, for an average prevalence rate of 0.456, with a stable baseline incidence initiation of 0.025 (or 2.5 absolute percentage points) [6, 53]. For evaluating the intervention, we expect to have approximately 1273 patients with clinic visits per year with site-specific numbers estimated as 227, 215, 135, 104, 403, and 189. Power was estimated using simulations making conservative assumptions about the number of available appointments, and rates of increase. From simulations, we have > 80% power to detect increased rates of initiation resulting in average prevalence after year 2 (1^st^ year of intervention) of 48.2% for the control practices vs. 57.0% for the intervention practices, an absolute difference of 8.7%, which is less than the 10% difference we expect to observe. In terms of the logistic regression model, this equates to a 6% relative increase per year in rates of incident initiation (for a secular trend with an absolute increase of 2.5%) in the control group and a 24.3% relative increase in the intervention group. Subgroup analyses: For a subgroup of half our sample (e.g., stratifying by sex, age at visit, or race/ethnicity) we have 80% power to detect a difference in rates of 49% vs. 61%.

#### Implementation trial

*Quantitative analysis.* Descriptive statistics (e.g., frequency, percentage mean, standard deviation [SD], median, range) will be used to summarize quantitative measures. Scores for Provider HPV Vaccine-related Knowledge/Practices will be modeled at the provider level using mixed models similarly to the methods for the Effectiveness Trial, to properly account for repeated observations and within-practice clustering, but with a continuous rather than binary outcome. Model-based predictions with confidence intervals will be reported over time and by intervention exposure, and will be displayed graphically. We expect to be underpowered for formal hypothesis testing, but model-based effect estimates for HPV-related Knowledge and Practices will be reported. *Sample Size and Power.* We recognize that we may not have sufficient power to test objective measures of provider HPV vaccine-related knowledge and practices, or objective ratings of the intervention; as such, the Implementation Trial is a secondary aim, and the provider knowledge/practices portion of the analysis will be exploratory.

*Qualitative analysis.* We will code interview transcripts and analyze them using content analysis. Two investigators will evaluate each transcript using line-by-line open coding to develop a preliminary codebook of emerging themes. We will use the preliminary codebook to guide additional analyses, and will develop a robust codebook as additional transcripts are analyzed. We will use team discussions to arrive at a consensus regarding final coding, aggregate the codes, and categorize them to identify major themes. Data saturation will occur when no new themes emerge. Qualitative software (e.g., NVivo) will be used for analysis.

*Mixed methods: Integration of data*. Results from the quantitative and qualitative analyses will be integrated through comparison to generate metainferences regarding the feasibility/acceptability of the intervention. The analysis will be weighted toward the qualitative strand [54]. The mixed analysis will expand our understanding of providers’ experiences implementing the intervention.

### Data management and safety monitoring plan

Participation in the research aspects (i.e., surveys and interviews) of this clinical trial are optional for healthcare providers. Intervention Champions will not have access to identifiable provider data and will not know which healthcare providers from their site choose to participate or not participate in the research aspects of the study. Nevertheless, it is possible that some healthcare providers who do not wish to participate in the research aspects of the study may perceive pressure to do so and may believe that not participating would be viewed negatively by their peers or superiors. Therefore, a data safety monitoring plan will be implemented to monitor for Unanticipated Problems related to research participation. The study PIs, and the Intervention Champions at each site, will be responsible for monitoring protocol conduct across all participating sites and reporting any deviations or unanticipated problems related to the protocol intervention and/or research procedures performed during this study to the study sIRB (UAB-IRB). Reports of unanticipated problems will include a description of the circumstances surrounding the unanticipated problem and the PI assessment of its impact on the study’s overall risk–benefit ratio. If an unanticipated problem appears to significantly affect the study’s risk–benefit ratio, an urgent review of the unanticipated problem will be made to determine whether immediate action is required. Based on the frequency and severity of unanticipated problems, the study PIs may deem it necessary to suspend or terminate the trial. When appropriate, the study PIs may recommend that unanticipated problems be reported to federal agencies.

## Discussion

The overall goal of this study is to prevent HPV-related neoplasms and their associated morbidity and mortality in the vulnerable population of young cancer survivors. We will test the effectiveness of HPV PROTECT – an intervention aimed at improving HPV vaccination rates in young cancer survivors – that is targeted to healthcare providers in the pediatric oncology setting. To our knowledge, this is the first evidence-based, provider-directed intervention to be adapted for pediatric oncology to specifically target the low uptake of the HPV vaccine among young cancer survivors.

Our intervention includes components that have been tested and proven effective in the general pediatric population (i.e., provider communication training [17, 20, 32], provider assessment feedback/coaching [20–22, 31], and provider toolkit [22, 26]). Additionally, in adapting the HPV PROTECT intervention for pediatric oncology, we have incorporated evidence specific to the young cancer survivor population, in order to address issues and concerns that are particularly relevant to these young survivors and to the healthcare providers caring for them (e.g., importance of recommendations from oncology providers, appropriate time to re-initiate vaccinations post-therapy, number of doses needed for series completion) [6, 7].

Our approach is innovative in several ways. We have established a consortium of participating institutions to test the HPV PROTECT intervention. These geographically-diverse institutions are representative of pediatric oncology practices of varying sizes and characteristics serving diverse and medically-underserved minority populations, and are especially enriched by sites serving populations in areas of lowest HPV vaccine uptake (i.e., U.S. southern states) [49, 50]. Our investigative team brings together extensive clinical and research expertise (including expertise in implementation science), and our Intervention Champions at each site are well-engaged and immersed in their respective clinical programs and have ready-access to the populations of interest. Additionally, the hybrid clinical effectiveness-implementation trial design will allow us to determine the effectiveness of the provider-directed intervention, and to understand the mechanisms and processes underlying successful implementation of the intervention in the pediatric oncology setting. Finally, we will collect and evaluate data from multiple perspectives, including clinic-level objective vaccination rates, intervention fidelity measures, provider knowledge and practices relevant to HPV vaccination, and provider perspectives regarding intervention feasibility, acceptability, and appropriateness.

We also acknowledge some study limitations. We have addressed threats to internal validity of the study design through use of rigorous methodology that incorporates construction of cohorts inclusive of the entire targeted survivor population at each site. Nevertheless, it is possible that additional unmeasured effects, in addition to the intervention effect (e.g., a media campaign not related to the study) could affect vaccine uptake during the intervention and sustainability periods. Additionally, we recognize that it is possible that survivors who have clinic visits toward the end of each study year will have less time to receive their vaccinations. A sensitivity analysis will be conducted to address this issue. It is also possible that providers may not participate in the intervention activities, potentially affecting intervention fidelity. The Intervention Champions will track clinician engagement at their sites and provide coaching and additional opportunities for providers needing remediation. It is also possible that providers will consent to research participation but then fail to complete the required measures. To address this issue, study CRAs will monitor data collection in real-time and send reminders to participants who have not completed study measures, in order to optimize data collection.

Despite these limitations, this study addresses important gaps in knowledge relevant to the prevention of HPV-related malignancies in young cancer survivors. We will test the effectiveness of an evidence-based provider-directed intervention, adapted for the pediatric oncology setting, to increase HPV vaccine initiation in young cancer survivors receiving care in pediatric oncology clinics. We will also procure information regarding intervention delivery to inform future implementation efforts. If proven effective, HPV PROTECT will be readily disseminable for testing in the larger pediatric oncology community to increase HPV vaccine uptake in cancer survivors, facilitating protection against HPV-related morbidities for this vulnerable population.

## Trial status

UAB IRB Protocol Number: 300005305, initially approved 26 Jul 2020. Recruitment began 1 Feb 2021. Current protocol version: Amendment 2, 04/20/2022, approved 03 May 2022. Anticipated study completion date: 31 Jan 2026.

## Data Availability

The study principal investigators, CRA at the site coordinating center, and statistician will have full access to the final data set. De-identified quantitative data underlying the published study results, with codebook, will be made available to researchers who provide a methodologically sound proposal to the study PIs, released under Creative Commons Attribution 4.0 Generic License (or equivalent), for up to 5 years following study completion.

## Abbreviations

CAR: Chimeric antigen receptor
COVID-19: Coronavirus disease of 2019
CRA: Clinical research assistant
EMR: Electronic medical record
HIPAA: Health information portability and accountability
HPV: Human papillomavirus
IRB: Institutional review board
PCP: Primary care provider
PHI: Personal health information
PI: Principal investigator
QI: Quality improvement
RE-AIM: Reach, effectiveness, adoption, implementation, maintenance
REDCap®: Research Electronic Data Capture
sIRB: Single institutional review board
SMART: Streamlined, multisite, accelerated resources for trials
Tdap: Tetanus, diphtheria, and pertussis
UAB: University of Alabama at Birmingham
U.S.: United States
